# Supplementary figures and images for: Physics-informed graph neural networks for robust cross-patient epileptic seizure prediction via chimera state detection
Source: PLoS One. 2026 Apr 2;21(4):e0345470. doi: 10.1371/journal.pone.0345470 (PMC13046251; doi:10.1371/journal.pone.0345470)

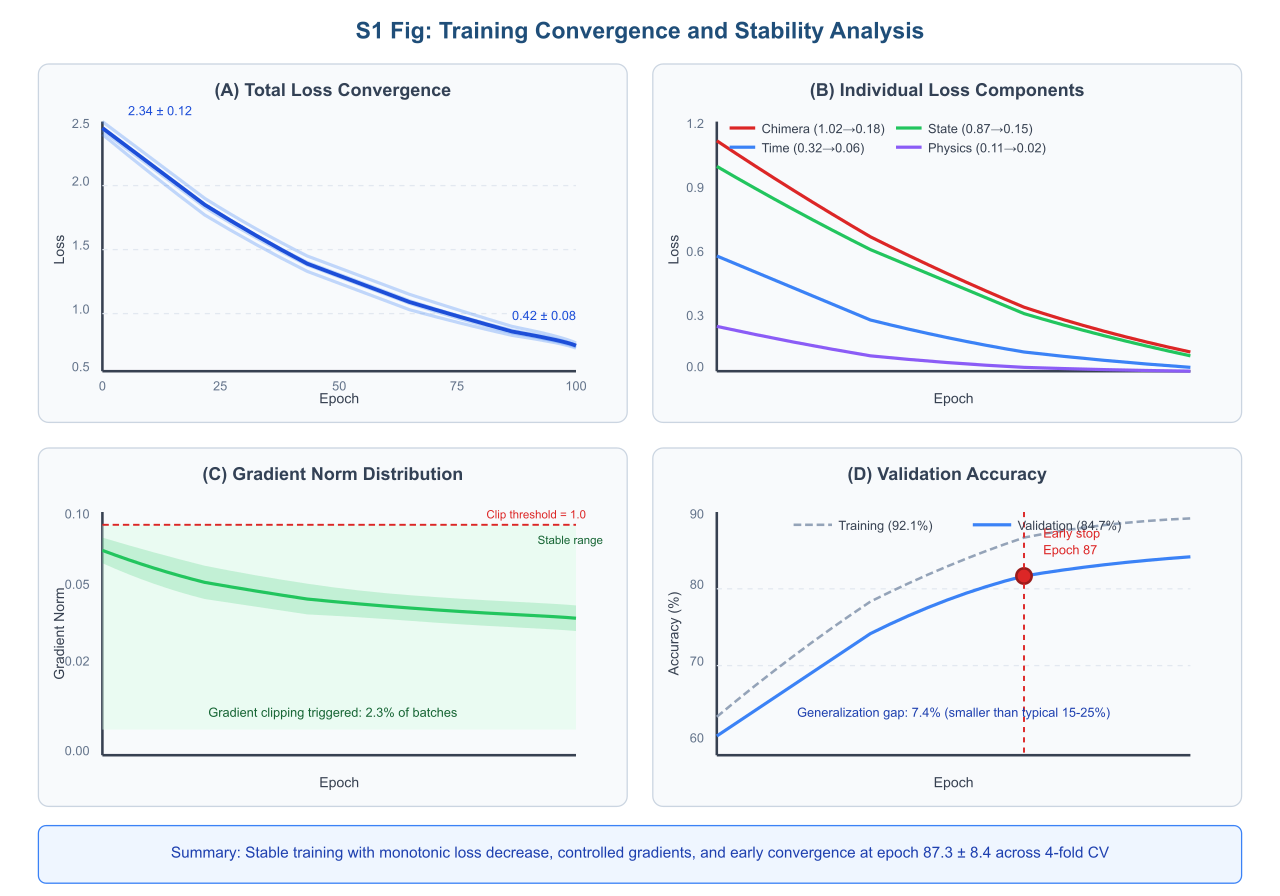

Supplement: S1 Fig — Four-panel figure showing: (a) total loss convergence across epochs for all cross-validation folds, (b) individual loss component trajectories, (c) gradient norm distribution during training, and (d) validation accuracy progression with early stopping markers. (TIFF) [file pone.0345470.s001.tiff]

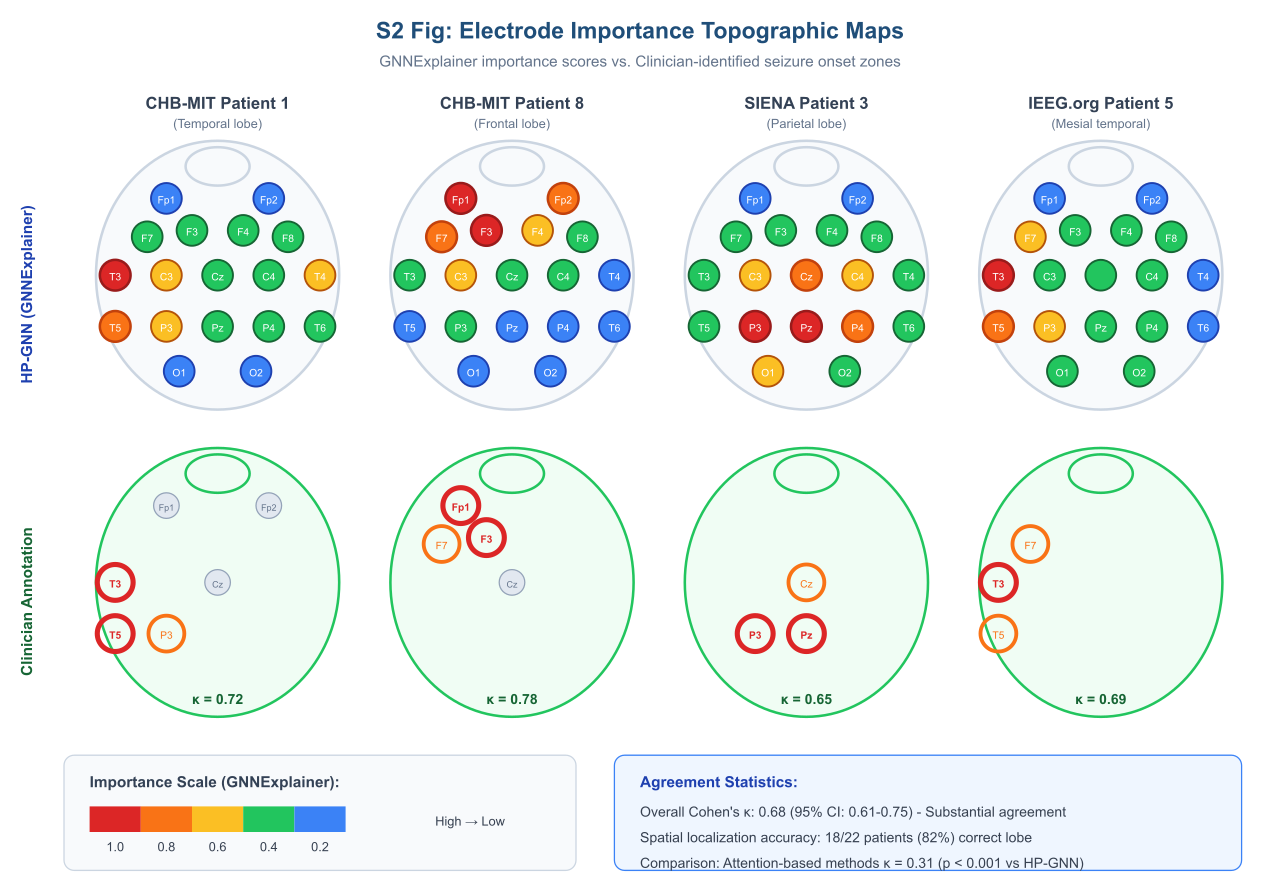

Supplement: S2 Fig — GNNExplainer-generated importance scores displayed on standard 10–20 montage for representative patients from each dataset, with comparison to clinician-annotated seizure onset zones and inter-rater agreement statistics. (TIFF) [file pone.0345470.s002.tiff]
