# Supplementary material for: Physics-informed graph neural networks for robust cross-patient epileptic seizure prediction via chimera state detection
Source: PLoS One. 2026 Apr 2;21(4):e0345470. doi: 10.1371/journal.pone.0345470 (PMC13046251; doi:10.1371/journal.pone.0345470)
Supplement: Attachment — Submitted filename: Response_Complete.docx [file pone.0345470.s005.docx]

**Point-by-Point Response to Reviewers**

Manuscript ID: PONE-D-25-65954

*Physics-Informed Graph Neural Networks for Robust Cross-Patient Epileptic Seizure Prediction via Chimera State Detection*

We sincerely thank the Academic Editor and both Reviewers for their constructive comments. We have carefully addressed each point with detailed responses and specific manuscript changes below.

**JOURNAL REQUIREMENTS**

**Requirement 1: Style Requirements**

*Please ensure that your manuscript meets PLOS ONE's style requirements, including those for file naming.*

**Response:**

We have reformatted the entire manuscript according to PLOS ONE style guidelines. All files have been renamed (Fig1.tif, Fig2.tif, Table.docx, etc.).

**Requirement 2: Data Source Citations**

*Please ensure that your data sources are appropriately defined in the text, ensuring it is clear how access to the data may be acquired.*

**Response:**

We have updated all dataset citations with proper DOIs and access information.

**Requirement 3: Acceptable Data Repository**

*The repository you have noted in your Data Availability statement does not qualify as an acceptable data repository according to PLOS's standards. Please upload the minimal data set necessary to replicate your study's findings to a stable, public repository (such as figshare or Dryad).*

**Response:**

We have uploaded all replication materials to Figshare (PLOS-approved repository):

• Complete HP-GNN source code

• Trained model weights (all folds)

• Preprocessing pipeline scripts

• Minimal replication dataset (5 patients)

• Evaluation scripts with expected outputs

Data Availability Statement

The datasets used in this study are available as follows:

1. CHB-MIT Scalp EEG Database: Publicly available through PhysioNet at https://physionet.org/content/chbmit/1.0.0/ under the Open Data Commons Open Database License v1.0.

2. SIENA Scalp EEG Database: Publicly available through PhysioNet at https://physionet.org/content/siena-scalp-eeg/1.0.0/.

3. IEEG.org Database: Available upon institutional Data Use Agreement at https://www.ieeg.org.

4. Code and Replication Materials: All source code, trained model weights, preprocessing scripts, evaluation code, and a minimal replication dataset are available with supplementary material.

**Requirement 4: Supporting Information Captions**

*Please include captions for your Supporting Information files at the end of your manuscript.*

**Response:**

We have added comprehensive captions for all Supporting Information files.

**SUPPORTING INFORMATION:**

SUPPORTING INFORMATION

S1 Fig. Training convergence analysis. Four-panel figure showing: (a) total loss convergence across epochs for all cross-validation folds, (b) individual loss component trajectories, (c) gradient norm distribution during training, and (d) validation accuracy progression with early stopping markers.

S2 Fig. Electrode importance topographic maps. GNNExplainer-generated importance scores displayed on standard 10-20 montage for representative patients from each dataset, with comparison to clinician-annotated seizure onset zones and inter-rater agreement statistics.

S1 Code. Complete implementation package. Python source code including HP-GNN model architecture (PyTorch), training pipeline, data preprocessing scripts, evaluation metrics, and usage examples with documentation.

**Requirement 5: ZIP File Format**

*We are unable to open your Supporting Information file 'code.zip'. Please kindly revise and re-upload.*

**Response:**

We apologize for the technical issue. The file has been recompressed using standard ZIP format without password protection. We verified successful extraction on Windows 10/11, macOS Ventura, and Ubuntu 22.04. Large model files have been split into smaller archives (<25MB). A comprehensive README.txt is included.

**REVIEWER #1 COMMENTS**

**Comment 1.1: Research Question in Abstract**

*The paper is well written; however, the research question should be introduced and explained more clearly, especially in the abstract.*

**Response:**

We thank the reviewer for this feedback. We have revised the abstract to explicitly and prominently state the research question.

**Research Question**

Research Question: Can integrating physics-based constraints from Kuramoto oscillator theory with graph neural networks enable automated, robust, and interpretable chimera-based seizure prediction that generalizes across patients?

**Comment 1.2: Dataset and Preprocessing Details**

*Section 2.1 (Dataset and preprocessing) needs significant improvement: More details about the subjects and the data collection conditions should be provided. Additionally, the details of preprocessing, such as the order of preprocessing stages, the number of ICA components, the selection criteria to identify noisy components, and the number of noisy components, must be reported.*

**Response:**

We have substantially expanded Section 2.1 with all requested details.

**Subject Details (Section 2.1):**

Subject Demographics and Data Collection

Table 2 summarizes the demographic characteristics across all three datasets. The CHB-MIT database comprises pediatric patients (age range 1.5-22 years, mean 11.3 ± 5.2 years) recorded at Boston Children's Hospital using a standard clinical EEG system. The IEEG.org dataset includes adult patients (age range 18-58 years, mean 34.6 ± 12.8 years) with intracranial electrode implants for pre-surgical evaluation. The SIENA database contains adult patients (age range 21-65 years, mean 41.2 ± 15.3 years) recorded at the University of Siena, Italy, using a Micromed SystemPlus acquisition system with a sampling rate of 512 Hz and 29 channels in the extended 10-20 system.

Table 2 summarizes the demographic characteristics across all three datasets.

| **Dataset** | **N** | **Age (years)** | **Sex (F/M)** | **Seizure Types** | **Recording Hours** |
| --- | --- | --- | --- | --- | --- |
| CHB-MIT | 22 | 1.5-22 (11.3±5.2) | 17/5 | Focal, Generalized | 844 |
| IEEG.org | 16 | 18-58 (34.6±12.8) | 9/7 | Focal (intracranial) | 312 |
| SIENA | 14 | 21-65 (41.2±15.3) | 8/6 | Focal aware/impaired | 186 |

The diversity in age groups, seizure types, and recording systems across datasets enables comprehensive evaluation of cross-patient generalization.

**Preprocessing Pipeline (Section 2.1):**

Preprocessing Pipeline

All EEG data underwent a standardized preprocessing pipeline implemented in Python using MNE-Python. The preprocessing stages were applied in the following order:

Step 1 - Resampling: All recordings were resampled to a uniform 256 Hz using scipy.signal.resample_poly with a Hamming window to prevent aliasing artifacts.

Step 2 - Bandpass Filtering: A 4th-order Butterworth bandpass filter (1-50 Hz) was applied using zero-phase filtering (scipy.signal.filtfilt) to remove DC drift and high-frequency noise while preserving physiologically relevant frequency bands.

Step 3 - Independent Component Analysis (ICA): FastICA decomposition was performed with 20 components (random seed=42, tolerance=10⁻⁴, maximum iterations=200). Artifactual components were identified using five automated criteria: (1) kurtosis > 5.0 indicating eye blinks or muscle artifacts, (2) high-frequency power ratio > 40% (power above 30 Hz relative to total) indicating EMG contamination, (3) frontal topography with > 70% weight on Fp1/Fp2 electrodes indicating ocular artifacts, (4) low temporal autocorrelation < 0.3 at 1-second lag indicating random noise, and (5) correlation > 0.8 with EOG reference channels when available. Components meeting any criterion were flagged for removal. On average, 3.2 ± 1.1 components per patient (range: 1-6) were removed.

Step 4 - Normalization: Channel-wise z-score normalization was applied using statistics computed exclusively from interictal periods to prevent information leakage from ictal segments.

**Comment 1.3: Dataset Size and Generalizability**

*The primary dataset (CHB-MIT) includes data from 22 patients, which is relatively small. This limits confidence in generalizability across age groups, seizure types, and clinical settings. The authors should extend the validation using a larger dataset or an additional dataset.*

**Response:**

We have added validation on a third dataset (SIENA) and performed comprehensive subgroup analyses.

**SIENA Dataset (Section 2.1):**

SIENA Scalp EEG Database

To address concerns about generalizability across adult populations and different clinical settings, we performed additional validation on the SIENA Scalp EEG Database [43]. This dataset contains recordings from 14 adult patients (mean age 41.2 ± 15.3 years, range 21-65 years, 8 female) with medically refractory focal epilepsy. Recordings were acquired at the Neurology and Clinical Neurophysiology Unit, University of Siena, Italy, using a Micromed SystemPlus system with 29 channels in the extended 10-20 configuration at 512 Hz sampling rate. This dataset provides independent validation across: (1) different geographical regions (Europe vs. North America), (2) different acquisition systems (Micromed vs. clinical EEG), (3) different patient demographics (European adult vs. North American pediatric), and (4) different annotation protocols.

**Table Cross-Dataset Performance – Added to Section 3.1 (Results):**

Table 7 demonstrates consistent performance across all three datasets, with modest variation (5.5% range) substantially smaller than the 20-35% degradation typical of purely data-driven approaches.'

| **Dataset** | **Accuracy** | **95% CI** | **Sensitivity** | **FP/hr** |
| --- | --- | --- | --- | --- |
| CHB-MIT | 84.7% | 82.3-87.1% | 89.3% | 0.48 |
| IEEG.org | 79.2% | 75.8-82.6% | 83.1% | 0.62 |
| SIENA | 82.3% | 79.1-85.5% | 86.7% | 0.54 |

The robust performance across diverse patient populations, acquisition systems, and clinical settings supports the generalizability of our approach.

**References (2023-2025):**

• Detti P, de Lara GZM, Bruni R, et al. Siena Scalp EEG Database (version 1.0.0). PhysioNet. 2023. https://doi.org/10.13026/5d4a-j060

**Comment 1.4: Computational Cost and Training Stability**

*Since the proposed architecture is very complex and the training dataset is small, more discussion supported by quantitative analysis on computational cost and training stability would be necessary.*

**Response:**

We have added a comprehensive new subsection analyzing computational requirements and training stability.

**Section 2.6.1 (new subsection):**

2.6.1 Computational Analysis and Training Stability

Given the architectural complexity of HP-GNN and the relatively limited training data, we conducted comprehensive analysis of computational requirements and training stability to ensure reproducibility and practical deployment feasibility.

Computational Resources

All experiments were conducted on a workstation equipped with an NVIDIA A100 GPU (40GB VRAM), AMD EPYC 7742 CPU (64 cores), and 256GB RAM. Table 3 summarizes the computational requirements for HP-GNN compared to baseline methods.

**Computational Comparison:**

Table 3 summarizes the computational requirements for HP-GNN compared to baseline methods.'

| **Method** | **Parameters** | **Training (h/fold)** | **Inference (ms)** | **GPU Memory** |
| --- | --- | --- | --- | --- |
| HP-GNN (Ours) | 2.47M | 4.2 ± 0.3 | 12.3 ± 1.2 | 8.4 GB |
| Transformer | 8.92M | 6.1 ± 0.4 | 45.2 ± 3.1 | 12.8 GB |
| ResNet-18 | 11.2M | 3.5 ± 0.2 | 15.4 ± 1.4 | 6.2 GB |
| BiLSTM | 3.14M | 2.8 ± 0.2 | 8.7 ± 0.8 | 4.1 GB |
| TCN | 4.21M | 3.1 ± 0.2 | 14.1 ± 1.2 | 5.3 GB |

HP-GNN achieves real-time inference capability (>80 windows/second at 256 Hz), enabling practical deployment in clinical monitoring systems.

**Section 2.6.1:**

Training Stability Analysis

To ensure reliable convergence despite the complex architecture, we monitored multiple stability metrics throughout training:

Loss Convergence: The total loss decreased monotonically from 2.34 ± 0.12 (epoch 1) to 0.42 ± 0.08 (epoch 100) across all cross-validation folds, with no oscillations or divergence observed. Individual loss components showed balanced optimization: chimera detection loss (1.02 → 0.18), state classification loss (0.87 → 0.15), time regression loss (0.32 → 0.06), and physics regularization loss (0.11 → 0.02).

Gradient Flow: Mean gradient magnitudes remained within the stable range [0.001, 0.1] throughout training, with standard deviation < 0.02. Gradient clipping (max norm = 1.0) was triggered in only 2.3% of training batches, predominantly during the first 10 epochs.

Early Stopping: Validation accuracy convergence occurred at epoch 87.3 ± 8.4 across folds, well before the 100-epoch maximum, indicating that the model learned meaningful representations without overfitting.

Generalization Gap: The difference between training accuracy (92.1%) and validation accuracy (84.7%) was 7.4%, substantially smaller than the 15-25% gap typically observed in deep learning models trained on similar dataset sizes, suggesting that physics constraints provide effective regularization.

Cross-Fold Consistency: Coefficient of variation (CV) was below 10% for all performance metrics across the four cross-validation folds, demonstrating robust and reproducible results.

Detailed training curves are provided in Supplementary Figure S1.

**REVIEWER #2 COMMENTS**

**Comment 2.1: Comprehensive Literature Review**

*One major issue that I observed is the lack of proper comprehensive review of related works. It is expected the authors to include a separate section and thoroughly review the related work. Besides, majority of the references are outdated and very few recent works (e.g. 2023, 24, 25) are covered.*

**Response:**

We sincerely thank the reviewer for this critical feedback. We have added a dedicated Section 1.5 'Related Work and Positioning' with comprehensive coverage of recent literature (14 new references from 2023-2025).

**Section 1.5 (Related Work):**

1.5 Related Work and Positioning

We organize the related work into five thematic areas, emphasizing recent advances (2023-2025) that contextualize our contributions.

Deep Learning for Seizure Prediction

Recent years have witnessed significant advances in deep learning approaches for seizure prediction. Zhang et al. [27] developed a large-scale self-supervised pre-training framework using over 10,000 hours of EEG data, achieving 87.2% sensitivity on the CHB-MIT dataset. However, their approach showed 25% accuracy degradation when applied to new patients without fine-tuning, highlighting persistent generalization challenges. Song et al. [28] introduced the EEG Conformer architecture combining convolutional and transformer layers, achieving state-of-the-art results on multiple EEG classification benchmarks. Pinto et al. [29] provided a comprehensive review of deep learning architectures for seizure prediction, identifying cross-patient generalization and clinical interpretability as the two most critical remaining challenges. Tang et al. [30] proposed self-supervised graph neural networks that improved data efficiency by 40% through contrastive pre-training, though their approach remained limited to patient-specific models.

Graph Neural Networks for Neurophysiological Signals

Graph-based approaches have gained prominence for modeling brain connectivity. Cui et al. [31] introduced BrainNetFormer, applying graph transformers to brain networks for seizure prediction with attention-based interpretability. Liu et al. [32] developed temporal graph neural networks that explicitly model evolving connectivity patterns, achieving 81.3% accuracy on CHB-MIT with improved temporal resolution. Chen et al. [33] proposed multi-scale graph convolutional networks capturing both local and global connectivity patterns. Wang et al. [34] combined spatial attention mechanisms with temporal graph convolutions, demonstrating that explicit connectivity modeling outperforms treating EEG channels as independent time series. However, none of these approaches incorporated physics-based constraints, limiting their ability to leverage domain knowledge about neural dynamics.

Physics-Informed Machine Learning for Neuroscience

Physics-informed approaches have shown promise for scientific machine learning. Kumar et al. [35] extended this framework to neural oscillator models, showing that Kuramoto-inspired constraints improved seizure detection accuracy by 8% while reducing training data requirements by 30%. However, their approach used simplified pairwise interactions rather than higher-order hypergraph structures. Our work advances this direction by integrating Kuramoto dynamics with hypergraph neural networks, enabling capture of collective synchronization patterns involving multiple neural populations simultaneously.

Chimera States in Neuroscience

Experimental and computational evidence for chimera states in neural systems has accumulated rapidly. Majhi et al. [36] demonstrated chimera-like patterns in multilayer neural network models during transitions to seizure-like activity, providing theoretical support for chimera states as seizure precursors. Computational studies using detailed cortical models have shown that chimera states emerge naturally in networks with realistic connectivity and dynamics, with chimera index serving as a robust biomarker for pre-ictal states.

Multimodal and Explainable Approaches

Clinical deployment requires both robust performance and interpretability. Rahman et al. [37] demonstrated that multimodal EEG-ECG fusion improved seizure prediction sensitivity by 12% through capturing autonomic nervous system changes preceding seizures. Zhou et al. [38] developed XAI-Seizure, an explainable framework combining gradient-based and attention-based interpretability methods. Li et al. [39] applied GradCAM-based explanations to seizure forecasting, achieving 0.58 Cohen's κ agreement with epileptologists. Shoeibi et al. [40] provided a comprehensive review emphasizing that clinical adoption requires interpretability scores above 0.6 κ agreement with expert judgment. Our approach addresses this requirement through physics-grounded explanations that clinicians can validate against established neurophysiological principles.

In summary, while substantial progress has been made in individual areas, no existing approach simultaneously addresses automated chimera detection, cross-patient generalization, data efficiency, and clinical interpretability. HP-GNN uniquely integrates insights from all five research areas to provide a comprehensive solution.

**References (2023-2025):**

• [27] Zhang X, Yang Q, Wang T, et al. Large-Scale Self-Supervised Pre-Training for EEG-Based Seizure Forecasting. Neural Networks. 2024;169:312-325.

• [28] Song Y, Zheng Q, Liu B, Gao X. EEG Conformer: Convolutional Transformer for EEG Decoding and Visualization. IEEE Trans Neural Syst Rehabil Eng. 2023;31:710-719.

• [29] Pinto MF, Leal A, Dourado A, et al. Deep Learning for Seizure Prediction: Architectures, Datasets, and Future Directions. Neural Comput Appl. 2023;35:6413-6433.

• [30] Tang S, Dunnmon JA, Saab K, et al. Self-Supervised Graph Neural Networks for Improved Electroencephalographic Seizure Analysis. ICLR. 2024.

• [31] Cui H, Dai W, Zhu Y, et al. BrainNetFormer: Graph Transformer for Brain Network Analysis. Proc AAAI. 2023;37:14128-14136.

• [32] Liu Y, Chen X, Ward RK, Wang ZJ. Temporal Graph Neural Networks for Epileptic Seizure Prediction. IEEE Trans Biomed Eng. 2024;71(3):847-858.

• [33] Chen B, Li Q, Zhang M, Wang H. Multi-Scale Graph Convolutional Networks for Seizure Forecasting. Med Image Anal. 2024;92:103052.

• [34] Wang S, Zhou Y, Li X, et al. Attention-Based Spatial-Temporal Graph Neural Networks for EEG Analysis. J Neural Eng. 2024;21(2):026018.

• [35] Kumar A, Singh R, Sharma N. Oscillator Dynamics-Constrained RNNs for Seizure Detection. Biomed Signal Process Control. 2024;89:105724.

• [36] Majhi S, Rakshit S, Ghosh D. Chimera States in Multilayer Networks During Epileptic Transitions. Phys Rev E. 2023;108(4):044308.

• [37] Rahman MM, Sarkar AK, Hossain MA, et al. Multimodal EEG-ECG Seizure Prediction Using Deep Fusion. IEEE J Biomed Health Inform. 2024;28(3):1456-1467.

• [38] Zhou Y, Huang S, Xu W, et al. XAI-Seizure: Explainable Deep Learning for Seizure Prediction. Expert Syst Appl. 2024;238:121856.

• [39] Li F, Chen W, Zhao S, et al. GradCAM-Based Explainable Framework for Seizure Forecasting. Artif Intell Med. 2024;146:102698.

• [40] Shoeibi A, Ghassemi N, Khodatars M, et al. Epileptic Seizure Detection Using Deep Learning: A Review. Int J Environ Res Public Health. 2023;20:4517.

**Comment 2.2: Presentation Quality**

*Presentation needs improvement; most images and graphs are of low quality; there are some grammatical and typos; too many short subsections exist, especially in section 1. The affiliations seem to be wrong.*

**Response:**

We have comprehensively addressed all presentation issues.

**1. Image Quality: All figures were corrected.**

**2. Grammatical Corrections:**

| **Location** | **Original** | **Corrected** |
| --- | --- | --- |
| Abstract, line 5 | minutes.However | minutes. However |
| Section 2.1, line 3 | anti- aliasing | anti-aliasing |
| Section 2.1, line 8 | range 1- 18 | range 1-18 |
| Section 2.2, line 12 | window=\'hamming\' | window='hamming' |
| Section 3.2, line 4 | patienthours | patient-hours |

**3. Affiliations:**

**Correct Affiliations:**

Masoud Amiri¹*, Bahador Makkiabadi¹

¹Department of Biomedical Engineering, School of Medicine, Kermanshah University of Medical Sciences, Kermanshah, Iran.

*Corresponding author: masd.amiri@yahoo.com

**4. Section Structure Reorganization:**

Introduction subsections have been merged:

• Sections 1.1 + 1.2 → 'section 1.1: Clinical Background and Evolution of Seizure Prediction'

• Sections 1.3 + 1.4 → 'section 1.2: Deep Learning Revolution and Chimera States'

• Section 1.5 (NEW) → 'section 1.3: Related Work and Positioning'

• Section 1.6 (NEW)→ 'section 1.4: Novel Contributions'

• Section 1.7 → 'section 1.5: Our Approach'

**Comment 2.3: Novelty and State-of-the-Art Advances**

*The novelty and advances to the state of the art should be clarified and quantified, as it is not very clear in the current version.*

**Response:**

We have added Section 1.6 explicitly quantifying each contribution.

**Section 1.6 (New):**

1.6 Novel Contributions and Quantified Advances

This work makes five distinct contributions, each with quantified improvements over existing approaches:

Contribution 1: Physics-Informed Hypergraph Architecture

We introduce the first integration of Kuramoto oscillator dynamics with hypergraph neural networks for EEG analysis. Unlike prior GNN approaches using pairwise edges, our hypergraph formulation captures higher-order synchronization patterns essential for chimera detection.

Quantified Advance: +4.9% accuracy improvement over state-of-the-art Temporal-GNN (84.7% vs. 79.8%, p<0.001, Cohen's d=0.89).

Contribution 2: Automated Chimera Detection

HP-GNN automates chimera detection that previously required 8-40 hours of manual parameter tuning per patient, enabling practical clinical deployment.

Quantified Advance: +9.2% accuracy improvement over manual Delay Differential Analysis while eliminating expert tuning requirements (84.7% vs. 75.5%, p<0.001).

Contribution 3: Cross-Patient Generalization

Physics constraints provide an inductive bias that dramatically improves transfer to unseen patients, addressing a critical barrier to clinical deployment.

Quantified Advance: +14.6% cross-patient accuracy improvement over BiLSTM baselines (79.8% vs. 65.2%, p<0.001, Cohen's d=1.24).

Contribution 4: Data Efficiency

By constraining the solution space to physically plausible dynamics, HP-GNN achieves target performance with substantially less training data.

Quantified Advance: 35% reduction in training data requirements to achieve 80% accuracy (260 vs. 400 patient-hours).

Contribution 5: Clinical Interpretability

GNNExplainer combined with learned Kuramoto parameters provides explanations that clinicians can validate against neurophysiological knowledge.

Quantified Advance: κ=0.68 agreement with epileptologists versus κ=0.31 for attention-based methods (p<0.001); clinician trust rating 4.3/5.0 versus 2.8/5.0 for black-box models.

**Summary of Contributions:**

Table 1 summarizes our contributions with statistical significance.

| **Contribution** | **Metric** | **Improvement** | **p-value** |
| --- | --- | --- | --- |
| 1. Physics-Informed Hypergraph | Accuracy vs. T-GNN | +4.9% (84.7 vs 79.8) | <0.001 |
| 2. Automated Chimera | Accuracy vs. DDA | +9.2% (84.7 vs 75.5) | <0.001 |
| 3. Cross-Patient | Accuracy vs. LSTM | +14.6% (79.8 vs 65.2) | <0.001 |
| 4. Data Efficiency | Hours for 80% | 260 vs 400 (-35%) | N/A |
| 5. Interpretability | Cohen's κ | 0.68 vs 0.31 | <0.001 |

All improvements are statistically significant with Bonferroni correction for multiple comparisons.

**Comment 2.4: Undefined Variables in Equations**

*Some equations contain undefined variables like Eq. 2, 3.*

**Response:**

We have added comprehensive variable definitions after Equations 2 and 3.

**Define variables:**

where the variables are defined as follows:

State Variables:

• h(t) ∈ ℝᵈ: Hidden state vector at time t, representing the internal memory of the system (d=256 in our implementation)

• h'(t) ∈ ℝᵈ: Continuous-time derivative of the hidden state, governing state evolution dynamics

• y(t) ∈ ℝ²⁵⁶: Output embedding vector fed to the multi-task prediction heads

Input Variables:

• zₜ ∈ ℝ²⁵⁶: Input embedding from the hypergraph encoder at time step t, containing spatial connectivity information

State Matrices (Input-Dependent):

• A(zₜ) ∈ ℝᵈˣᵈ: State transition matrix controlling how the current state influences its derivative

• B(zₜ) ∈ ℝᵈˣ²⁵⁶: Input projection matrix determining how new inputs are incorporated into the state

• C(zₜ) ∈ ℝ²⁵⁶ˣᵈ: Output projection matrix mapping hidden states to outputs

• D(zₜ) ∈ ℝ²⁵⁶ˣ²⁵⁶: Skip connection matrix allowing direct input-to-output pathways

All state matrices are computed through learned linear projections of the input embedding:

A(z) = Wₐz + bₐ, B(z) = W_bz + b_b, C(z) = W_cz + b_c, D(z) = W_dz + b_d

where Wₐ, W_b, W_c, W_d and bₐ, b_b, b_c, b_d are learnable weight matrices and bias vectors, respectively. This input-dependent parameterization enables selective information retention based on content, distinguishing Mamba from traditional state space models with fixed dynamics.

For efficient computation, the continuous-time system is discretized using zero-order hold with step size Δ, converting differential equations to recurrence relations suitable for sequential processing.

**Comment 2.5: Additional Baseline Comparisons**

*It would be really beneficial to compare the proposed method with additional deep learning methods rather than GNN-based only. Also, compare the performance with ML techniques.*

**Response:**

We have expanded Table 5 to include classical ML and additional DL methods.

Table 5 presents comprehensive performance comparison across classical machine learning, deep learning, and graph neural network approaches.

**Classical Machine Learning Methods:**

| **Method** | **Accuracy** | **Sensitivity** | **Specificity** | **FP/hr** | **Cross-Patient** |
| --- | --- | --- | --- | --- | --- |
| Random Forest | 68.4±4.8% | 72.1±4.3% | 64.7±5.1% | 0.89 | 61.2±6.1% |
| SVM (RBF) | 71.2±4.3% | 74.8±4.1% | 67.6±4.8% | 0.82 | 63.7±5.8% |
| XGBoost | 73.6±3.9% | 77.3±3.7% | 69.9±4.2% | 0.76 | 65.2±5.4% |

**Deep Learning Methods:**

| **Method** | **Accuracy** | **Sensitivity** | **Specificity** | **FP/hr** | **Cross-Patient** |
| --- | --- | --- | --- | --- | --- |
| 1D CNN | 78.3±3.5% | 81.7±3.2% | 74.9±3.8% | 0.63 | 67.8±4.9% |
| BiLSTM | 77.2±3.6% | 80.4±3.3% | 74.0±3.9% | 0.67 | 65.2±5.2% |
| ResNet-18 | 79.7±3.2% | 83.1±2.9% | 76.3±3.5% | 0.59 | 69.3±4.6% |
| Transformer | 80.4±3.4% | 84.2±3.0% | 76.6±3.7% | 0.57 | 70.1±4.8% |
| TCN | 81.2±3.1% | 85.1±2.8% | 77.3±3.4% | 0.54 | 71.4±4.5% |

**Graph Neural Network Methods:**

| **Method** | **Accuracy** | **Sensitivity** | **Specificity** | **FP/hr** | **Cross-Patient** |
| --- | --- | --- | --- | --- | --- |
| GraphSAGE | 76.8±3.4% | 79.2±3.1% | 74.4±3.7% | 0.71 | 66.8±5.1% |
| GAT | 78.4±3.2% | 81.4±2.9% | 75.4±3.5% | 0.65 | 68.1±4.8% |
| Temporal-GNN | 79.8±3.0% | 82.7±2.8% | 76.9±3.3% | 0.61 | 69.5±4.6% |
| HP-GNN (Ours) | 84.7±2.1% | 89.3±1.9% | 80.1±2.4% | 0.48 | 79.8±3.2% |

HP-GNN significantly outperformed all baselines (paired t-test, p<0.001 with Bonferroni correction). The improvement was particularly pronounced for cross-patient generalization (+8.4% over TCN, +10.3% over Temporal-GNN), demonstrating the effectiveness of physics-informed constraints for domain transfer.

**Comment 2.6: Explainability Discussion**

*The authors emphasise on the explainability feature of the proposed method while very little (or no) discussion provided to address this in the results section.*

**Response:**

We extended Section 3.6 with comprehensive interpretability analysis.

**Section 3.6:**

3.6 Clinical Interpretability and Validation

A critical requirement for clinical deployment is the ability to explain model predictions in terms that clinicians can validate against neurophysiological knowledge. We evaluated HP-GNN's interpretability at three complementary levels.

Level 1: Electrode Importance via GNNExplainer

We applied GNNExplainer to identify which electrodes contributed most strongly to each prediction (Detailed are provided in Supplementary Figure S2). For each seizure event, the method generates importance scores for all 23 electrodes, which can be visualized as topographic maps overlaid on the standard 10-20 montage.

To validate these explanations, three board-certified epileptologists (combined experience: 42 years) independently reviewed 20 held-out seizure events and rated the plausibility of model-highlighted electrodes based on seizure semiology and their clinical knowledge. Table 9 summarizes the agreement metrics.

**Interpretability Metrics:**

Table 9 summarizes the agreement metrics between HP-GNN explanations and clinical judgment.

| **Metric** | **HP-GNN Value** | **Comparison** |
| --- | --- | --- |
| Inter-rater reliability (ICC) | 0.74 (95% CI: 0.68-0.80) | Good agreement |
| Model-clinician agreement (κ) | 0.68 (95% CI: 0.61-0.75) | Substantial |
| Spatial localization accuracy | 18/22 patients (82%) | Correct lobe |
| Within-patient consistency | r = 0.84 ± 0.11 | Highly consistent |

The substantial agreement (κ=0.68) exceeds the 0.6 threshold recommended for clinical decision support systems [40].

**Section 3.6:**

Level 2: Learned Kuramoto Parameters

The physics-informed regularization forces the model to learn Kuramoto parameters (coupling strength K, natural frequencies ω, phase lag α) that can be directly interpreted in terms of neural oscillation dynamics. Table 10 shows the learned parameters and their biological interpretations.

**Kuramoto Parameters:**

Table 10 shows the learned Kuramoto parameters and their biological interpretations.'

| **Parameter** | **Learned Value** | **Biological Interpretation** |
| --- | --- | --- |
| ω_synchronized | 2.3 ± 0.5 Hz | Delta band (pathological slowing) |
| ω_desynchronized | 9.1 ± 1.3 Hz | Alpha band (normal rhythm) |
| Coupling K | 0.71 ± 0.14 | Within chimera regime [0.4-0.9] |
| Phase lag α | 0.28 rad (16°) | ~19 ms conduction delay |

Remarkably, these learned values align with known neurophysiology: the synchronized frequency matches pathological delta slowing observed in epileptogenic tissue, while the phase lag corresponds to expected axonal conduction delays between cortical regions.

**Section 3.6:**

Level 3: Clinical Validation Study

The same three epileptologists evaluated overall clinical utility using a structured questionnaire with 5-point Likert scales. Table 11 presents the results.

**Clinical Validation:**

Table 11 presents the clinical utility assessment results.'

| **Assessment Criterion** | **Mean ± SD** | **% Agreement** |
| --- | --- | --- |
| Plausibility of highlighted electrodes | 4.2 ± 0.6 | 85% |
| Consistency with seizure semiology | 3.9 ± 0.7 | 75% |
| Utility for clinical decision-making | 4.1 ± 0.6 | 80% |
| Trust in predictions given explanations | 4.3 ± 0.5 | 90% |

Critically, the trust rating (4.3/5.0) significantly exceeded that for black-box BiLSTM predictions (2.8/5.0, p<0.001, Cohen's d=1.89), demonstrating that physics-grounded explanations substantially improve clinician confidence in model outputs.

**Section 3.6:**

Comparison with Alternative Interpretability Methods

We compared HP-GNN's interpretability against attention-based explanations from the Transformer baseline. While attention weights provide some insight into temporal focus, they showed lower agreement with clinical judgment (κ=0.31 vs. 0.68) and were rated as less trustworthy by clinicians (2.6/5.0 vs. 4.3/5.0). The advantage of physics-informed explanations is that they are grounded in established neurophysiological principles (oscillator synchronization, conduction delays) that clinicians can directly validate, rather than abstract learned patterns.
